# On the Path to Optimal Alchemistry

**DOI:** 10.1007/s10930-023-10137-1

**Published:** 2023-08-31

**Authors:** Magnus Lundborg, Jack Lidmar, Berk Hess

**Affiliations:** 1 ERCO Pharma AB, 11439 Stockholm, Sweden; 2 Department of Physics, KTH Royal Institute of Technology, 10691 Stockholm, Sweden; 3 Department of Applied Physics, KTH Royal Institute of Technology, 10691 Stockholm, Science for Life Laboratory, Solna, Sweden

**Keywords:** Alchemical free energy calculations, Lambda path optimization, Accelerated weight histogram method

## Abstract

Alchemical free energy calculations have become a standard and widely used tool, in particular for calculating and comparing binding affinities of drugs. Although methods to compute such free energies have improved significantly over the last decades, the choice of path between the end states of interest is usually still the same as two decades ago. We will show that there is a fundamentally arbitrary, implicit choice of parametrization of this path. To address this, the notion of the length of a path or a metric is required. A metric recently introduced in the context of the accelerated weight histogram method also proves to be very useful here. We demonstrate that this metric can not only improve the efficiency of sampling along a given path, but that it can also be used to improve the actual choice of path. For a set of relevant use cases, the combination of these improvements can increase the efficiency of alchemical free energy calculations by up to a factor 16.

## Introduction

In alchemical free energy calculations the nature of the simulated particles is changed. The goal of classical alchemistry was to obtain more precious materials from cheaper ones. In alchemical free energy calculations the goal is to obtain the difference in free energy between systems of different composition or with different interactions. A single such free energy difference between different chemical states is not useful by itself. But when considering thermodynamic cycles, a closed loop of multiple of such free energy differences between multiple states, meaningful free energy differences can be computed, see e.g. [1]. The power of alchemical free energy calculations lies in the property that two (or more) well chosen alchemical transformations can often lead to much more efficient calculations than sampling the physical pathway between the states one is interested in. A typical example is the computation of the difference in binding free energy of two (or more) ligands to a protein by computing the difference in the free energy of mutating the ligand when bound to the protein and when in solution. This avoids sampling the, physical or unphysical, pathway of (un)binding the ligand and the protein. [2, 3]

Initially, the main use of alchemical free energy calculations was to parametrize and validate molecular mechanics force fields, in particular for proteins. [4–9] A common check is to compare the free energies of compounds in different solvents with experiment. Here the compound is (de)coupled from the solvent by scaling the interactions of the compound with the solvent. Over the last two decades drug binding free energies have become one of the main application areas of molecular simulations. [10–13] However, there are still few, if any, marketed drugs developed using molecular dynamics based binding free energy calculations. [14] Here one can distinguish absolute binding free energy calculations, which are in method similar to solvation free energy calculations but with additional restraints, and the relative free energy difference between binding of two different drugs to the same molecule or one drug to different molecules. Relative free energy differences are obtained using alchemical transformations of the ligand or binding partners. When the goal is to compute free energy differences of many rather similar molecules, this enables efficient free energy calculations by alchemical transformation of a few atoms for each edge in a graph of molecules.

### The $\lambda$-Coupling Approach

To perform an alchemical calculation, an alchemical handle on the system is needed. The standard approach here is to introduce a coupling parameter called $\lambda$ to the Hamiltonian. This is done such that the Hamiltonian *H* corresponds to alchemical state *A* at $\lambda =0$ and alchemical state *B* at $\lambda =1$:

1 $$\begin{aligned} H(\textbf{x};\lambda )_{\lambda =0} = H_A;\,\;\;\,H(\textbf{x};\lambda )_{\lambda =1} = H_B \end{aligned}$$

where $\textbf{x}$ are the positions and momenta of all particles in the system. It is important to note that this procedure leaves the choice of Hamiltonian for intermediate values of $\lambda$ completely free. Most methods for calculating free energy differences require that *H* follows a continuous or discrete path between $H_A$ and $H_B$. Some methods use only a small number of values of $\lambda$ and then the requirement is that there is sufficient overlap of the ensembles between neighboring $\lambda$ points. [15] How much overlap is needed depends on the method. This is discussed in e.g. [15, 16] and [17].

We have not yet discussed actual methods to compute free energy differences, but one can imagine that the choice of path can have a large effect on the efficiency of the calculation. In particular, minimizing the change of the distribution of the ensemble along $\lambda$ will tend to improve the efficiency of free energy calculations. Performing such a minimization is a non-trivial task. In addition, when a path is given, there is the question of how to parametrize it, or, equivalently, how to distribute points along the path in the case of discrete methods. That this is a relevant question can be shown by replacing $\lambda$ by $\lambda ' = f(\lambda )$ where *f* is a non-linear function which does not modify the end points. We retain the same path, but the “speed” along the path is changed. Unless the chosen method internally optimizes this speed, the choice of $\lambda$ parametrization will have a large effect on the efficiency of the calculation, as we will demonstrate later.

### The $\lambda$ Path

There are infinitely many paths to choose from. The question is what is an efficient and practical choice of path. The standard approach is to linearly interpolate the Hamiltonians of the end states. But when atoms disappear or appear at one of the end states, linear interpolation will give issues with singularities [18, 19] in both the Lennard-Jones and Coulomb potential, since nothing prohibits a particle from being placed on top of another. To avoid this, so called *soft-core* potentials are needed.

The most commonly used formulation of the soft-core potential originates from the molecular dynamics group of Herman Berendsen. This is a place where many of the early developments in the field have happened, not only because of ideas of Berendsen himself, but also because of the open and inspiring environment he created, which promoted sharing ideas and attracted visiting scientists. The basis of this soft-core setup was developed by Thomas Beutler [20] in the group of Wilfred van Gunsteren, who was Herman’s first PhD-student. This used separate modified $\lambda$-dependent distance functions for computing the Lennard-Jones and Coulomb potentials. Through the close contacts between the groups of Berendsen and Van Gunsteren, this idea was picked up by Berk Hess and improved by using the Lennard–Jones distance function also for the Coulomb interactions. The use of the same function for all components of the non-bonded interactions avoids artificial minima. Also the exponent *p* was changed from 2 to 1. This setup was implemented in the 3.0 version [21] of the GROMACS simulation package, which originates from the group of Herman Berendsen. The equations for a pair potential *V* are as follows:

2 $$\begin{aligned} \begin{aligned}\begin{aligned} V_{sc}(r)= & {} {(1-{\lambda })}V^A(r_A) + {\lambda }V^B(r_B) \\ r_A= & {} \left( \alpha \sigma _A^6 {\lambda }^p + r^6 \right) ^\frac{1}{6} \\ r_B= & {} \left( \alpha \sigma _B^6 {(1-{\lambda })}^p + r^6 \right) ^\frac{1}{6}\end{aligned}\end{aligned} \end{aligned}$$

where *r* is the distance between the particles involved and $\sigma$ is the Lennard–Jones length parameter. $V^A$ and $V^B$ are the unmodified Van der Waals, or electrostatic, potentials in state *A* and *B*, respectively, whereas $r_A$ and $r_B$ are the distances between the particles in the two states. The exponent *p* is usually set to 1. The parameter $\alpha$ is critical, as it determines the point where the excluded volume of atoms (dis)appears. Nowadays a value of 0.5 is recommended. These soft-core equations are now implemented in many molecular dynamics packages and widely used. This choice of path is by now more than two decades old, and other similar solutions were developed in the same period of time. [22] More recently, alternative soft-core potentials have been proposed. [23, 24]

## Free Energy Calculations

### Methods Sampling at Fixed Values of $\lambda$

One of the first methods used to estimate free energy differences is free energy perturbation. [25] Here an estimate of a free energy difference with a perturbation Hamiltonian is computed from an ensemble sampled at the unperturbed Hamiltonian. To achieve reasonable efficiency this requires that the ensemble overlap is relatively large. This limits the usefulness of perturbation.

The original method for computing alchemical free energies is thermodynamic integration. [26] This uses the fact that the free energy difference is equal to the integral of the gradient of the Hamiltonian over $\lambda$, i.e., $\frac{\partial H(\textbf{x};\lambda )}{\partial \lambda }$. The difference in Gibbs free energy *G* between states *B* and *A* is then:

3 $$\begin{aligned} G_B - G_A = \int _{0}^1 \left\langle \frac{\partial H(\textbf{x};\lambda )}{\partial \lambda }\right\rangle _\lambda d\lambda \end{aligned}$$

It is important to note that one has to provide an ensemble average of the gradient of the Hamiltonian at each value of lambda. In thermodynamic integration, simulations are performed at a series of fixed $\lambda$ values to obtain values of the ensemble average of the gradient. The free energy difference is then computed using numerical integration. Low-order methods like the trapezoidal rule are usually preferred due to the presence of statistical errors, although careful application of higher-order methods may be beneficial. [27, 28] But as the Hamiltonian often changes significantly and in a non-linear fashion along $\lambda$, quadrature errors tend to be large. The main question in setting up thermodynamic integration calculations is then how to choose the points along $\lambda$ to minimize the systematic quadrature error. The question of statistical efficiency has largely been ignored. But as thermodynamic integration has been superseded by methods that do not suffer from systematic errors, we will not further discuss this.

The Bennett Acceptance Ratio (BAR) method [15] uses the same setup of equilibrium simulations at several values of $\lambda$ as thermodynamic integration, but does not suffer from systematic errors in the limit of sufficient sampling. BAR uses Hamiltonian differences computed from two equilibrium simulations, each run at a different values of $\lambda$. By computing differences in both directions, an accurate estimate of the free energy can be computed, provided that the two distributions overlap. When the endpoints are well separated, intermediate $\lambda$-points need to be introduced to break up the free energy difference into smaller pieces. It has been proven that BAR provides the most efficient asymptotically unbiased estimate of the free energy difference between two states. [29] BAR can be slightly improved when using more than two $\lambda$-points by using Hamiltonian differences between all pairs of $\lambda$-points, this leads to the MBAR method. [30]

All methods mentioned here usually require several intermediate $\lambda$-points to efficiently compute free energies between the end states of interest. With a given path, the number of $\lambda$-points and their distribution need to be chosen. Over the years the community has realized that the choice of path can have a strong effect on the efficiency of free energy estimate. [31–33] Still, in practice points are often distributed uniformly and sometimes a higher density is used in regions that are more difficult to sample (usually at one of the end points). There have been quite a few efforts to systematically improve the efficiency by optimizing the distribution of distribution of $\lambda$-points, see e.g. [34–36]. There have been a few attempts to improve the path itself [31, 32], with more studies coming out in recent years, but all of those use metrics that do not take time correlations into account.

### The Accelerated Weight Histogram Method

The accelerated weight histogram method (AWH) [37, 38] operates within the general framework of expanded ensembles, [39] where the parameter $\lambda$ becomes dynamical. This way all $\lambda$-values are included in a single simulation, in proportion given by a specified target distribution $\pi _\lambda$. In the general case $\lambda$ can be multidimensional and include both alchemical degrees of freedom and physical reaction coordinates. For the present work we only consider one or more alchemical coordinates. The samples generated by the simulation will, when converged, follow a joint distribution

4 $$\begin{aligned} P(\textbf{x},\lambda ) = \frac{1}{\mathcal {Z}} \pi _\lambda e^{f_\lambda - \beta H(\textbf{x}; \lambda )}, \end{aligned}$$

where $\textbf{x}$ denotes all coordinates and momenta of the atoms. In the AWH algorithm [37, 38, 40] the hyperparameters $f_\lambda$ are adaptively fine-tuned until the marginal $P_\lambda = \int P(\textbf{x},\lambda ) d\textbf{x}$ matches the prescribed target $\pi _\lambda$, which translates to $f_\lambda \rightarrow \beta G_\lambda$ (up to a constant), where $G_\lambda$ is the (Gibbs) free energy at $\lambda$.

The GROMACS implementation [38, 40] uses two stages, where the first uses a heuristic but robust update procedure of the $f_\lambda$ to obtain a rough estimate. This is then followed by a second longer stage using an (asymptotically) optimal update, where the standard error decays like $1/\sqrt{N}$.

Because of its efficiency and the natural inclusion of a target distribution, we will use AWH as the main method in this work.

### Non-Equilibrium Methods

The issue of $\lambda$-parametrization also applies to non-equilibrium free energy methods based on Jarzynski’s equality [41] and Crooks’ fluctuation theorem [42]. Here, choices can be equally important, because the parametrization dictates the non-equilibrium protocol along the path and affects the friction, which in turn determines the amount of non-reversible work. As the statistical accuracy depends exponentially on the non-equilibrium work, reducing this work by improving the $\lambda$ parametrization has the potential to significantly improve the accuracy. When using Crooks’ fluctuation theorem, the statistical error is much smaller than with Jarzynski’s equality, but it still depends strongly on the friction.

The optimizations discussed below are highly relevant for non-equilibrium free energy methods, although we will not pursue this idea further here.

## Distance in Parameter Space

All the methods discussed above make use of a path in $\lambda$-space interpolating between the end points. In order to compare different paths and parametrizations, it is necessary to introduce some notion of distance on the parameter space [43]. Assuming the paths may be considered continuous, this can be done by specifying a metric tensor $g_{\mu \nu }(\lambda )$, where $\mu$ and $\nu$ refer to different dimensions when $\lambda$ is multidimensional. A useful metric may be obtained by relating it to the variance of the estimated free energy difference, $\textrm{Var}\ \overline{\Delta G}$. This is most easily done for the integration method, Eq. (3), when the ensemble averages are estimated by time averages, $\left\langle \partial H(\textbf{x};\lambda ) / \partial \lambda \right\rangle \approx (1/\tau _\lambda ) \int _0^{\tau _\lambda } (\partial H(\textbf{x}(t);\lambda ) / \partial \lambda ) dt$. Let us consider a continuous path $\{ \lambda ^\mu (s) | s \in [0,1] \}$ connecting the end states. Then

5 $$\begin{aligned} \textrm{Var}\ \overline{\Delta G} = 2 \int _0^1 \frac{{\dot{\lambda }}^\mu (s) g_{\mu \nu }(\lambda (s)) {\dot{\lambda }}^\nu (s)}{\tau _s} ds, \end{aligned}$$

where the dot denotes a derivative with respect to *s*, repeated Greek indices are summed over, and $\tau _s$ is the length of the simulation at $\lambda ^\mu (s)$. The metric is in this case

6 $$\begin{aligned} g_{\mu \nu }(\lambda ) = \int _0^\infty \left\langle \delta \mathcal {F}_\mu (t) \delta \mathcal {F}_\nu (0) \right\rangle _\lambda dt, \end{aligned}$$

where $\mathcal {F}_\mu = - \partial H(\textbf{x};\lambda ) / \partial \lambda ^\mu$ is a generalized force conjugate to $\lambda$ and $\delta \mathcal {F}_\mu = \mathcal {F}_\mu - \left<\mathcal {F}_\mu \right>_\lambda$, and it has been assumed that the correlation function appearing in the integrand is negligible for times larger than $\tau _s$. In practice separate simulations are carried out at distinct $\{ \lambda ^\mu (s_k) \}$, so that Eq. (5) will only be approximate due to discretization errors. These expressions also hold for BAR in the limit of tightly spaced $\lambda$-points, and for slowly driven non-equilibrium simulations in which *s* deterministically changes from 0 to 1. Indeed, the same metric also appears as a friction tensor in the excess work $W - \Delta G$ for slowly driven nonequilibrium processes, as derived in Ref. [44] using linear response theory. For this reason we will refer to Eq. (6) as the *friction metric*.

**Fig. 1:**
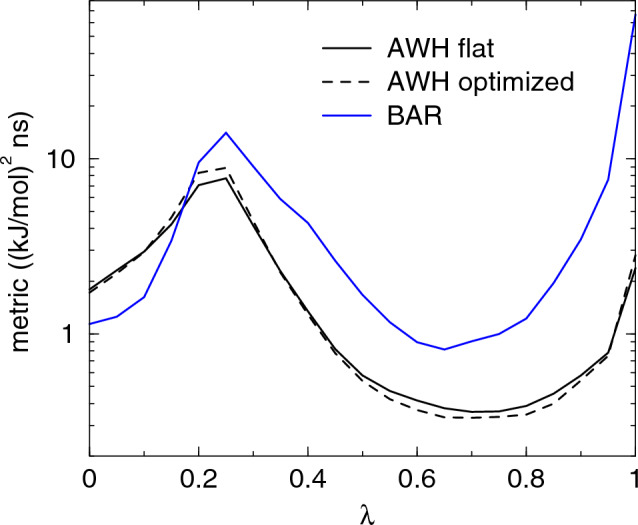
The diffusion metric, eq. (7), with AWH and friction metric, eq. (6) with (M)BAR for the hydration of the alanine dipeptide. The solid and dashed lines are the unoptimized and optimized metric, respectively. Note that the metric is plotted on log scale

For extended ensemble simulations like AWH, which carry out a diffusive random walk among the $\lambda$, a metric may instead be defined as [43]

7 $$\begin{aligned} g_{\mu \nu }(\lambda ) = \int _0^\infty \frac{\left\langle \delta \mathcal {F}_\mu (t) w_\lambda (t) \delta \mathcal {F}_\nu (0) w_\lambda (0) \right\rangle }{\left\langle w_\lambda ^2 \right\rangle } dt, \end{aligned}$$

where $w_\lambda (t) = P(\lambda | \textbf{x}(t))$, and the averages are taken with respect to the probability (4). Under a Markov assumption of the motion on $\lambda$-space, this metric may be directly related to the variance of $\overline{\Delta G}$ as in Eq. (5), with $\tau _s = \tau \pi _s$ equal to the fraction of total simulation time $\tau$ spent at *s*. Since the metric in Eq. (7) is proportional to the inverse diffusion tensor [43], we will refer to it as the *diffusion metric*. Numerically, it is quite close to the friction metric, but often somewhat smaller and with smoother variations (see Fig. 1 below).

The variance in Eq. (5) may be bounded from below, using the Cauchy-Schwarz inequality, as $\textrm{Var}\ \overline{\Delta G} \ge 2 \mathcal {L}^2 / \tau$, where $\mathcal {L}$ is the length of the path, [43]

8 $$\begin{aligned} \mathcal {L} = \int _0^1 \sqrt{{\dot{\lambda }}^\mu g_{\mu \nu }(\lambda ) {\dot{\lambda }}^\nu } ds. \end{aligned}$$

The bound is saturated when the samples are distributed as $\pi _s = \tau _s / \tau = \sqrt{{\dot{\lambda }}^\mu g_{\mu \nu }(\lambda (s)) {\dot{\lambda }}^\nu } / \mathcal {L}$ (or alternatively, the path is reparametrized such that $\pi _s = 1$).

For one-dimensional alchemical parameters $s = \lambda$ the formulas simplify to

9 $$\begin{aligned} \textrm{Var}\ \overline{\Delta G} = \frac{2}{\tau } \mathbb {V} = \frac{2}{\tau } \int _0^1 \frac{g(\lambda )}{\pi _\lambda } d\lambda \end{aligned}$$

and

10 $$\begin{aligned} \mathcal {L} = \int _0^1 \sqrt{g(\lambda )} d\lambda , \end{aligned}$$

while the optimal target distribution becomes $\pi _\lambda = \sqrt{g(\lambda )}/\mathcal {L}$. We introduced here $\mathbb {V} = \tau \textrm{Var}\ \overline{\Delta G} / 2 \ge \mathcal {L}^2$, which is a useful efficiency measure of the simulation. To quantify the reduction of the variance when going from a uniform distribution $\pi _\lambda = 1$ to the optimal, it is useful to define a (theoretical) improvement factor (IF), as $\text {IF} = \mathbb {V}_\textrm{opt} / \mathbb {V}_\textrm{unif} = \mathcal {L}^2 / \mathbb {V}_\textrm{unif}$. After equilibration, the statistical error decreases as the square root of time. Thus the improvement factor gives the reduction in computational time after equilibration. As mentioned, the expressions for the variance and distance discussed here are only approximate, because of discretization effects, Markov assumptions, or the restriction to slow $\lambda$-dynamics. For AWH, the variance estimate is, in addition, only asymptotic since it ignores nonequilibrium effects before it has fully converged. Nevertheless we expect that they will be useful in order to compare and optimize different paths and parametrizations. Importantly, the metrics defined here account for the time correlations in the dynamics, in contrast to the Fisher-Rao metric based on the equal time covariance $\left<\delta \mathcal {F}_\mu \delta \mathcal {F}_\nu \right>$. [43]

## Results

### Hydration Free Energy of the Alanine Dipeptide

The first test system is our beloved alanine dipeptide. But in this study we are not going to look at the phi-psi landscape, but rather at the free energy of hydration. Here we only consider the contribution of coupling molecule to water where we also couple the intramolecular interactions. To get the complete hydration free energy one needs to subtract the free energy for the edge of coupling the intramolecular interactions in vacuum, which is $-$318.5 kJ/mol. For both AWH and (M)BAR we used 21 equally spaced $\lambda$-points. The resulting free energy difference is rather large with $-$370.1 kJ/mol.

**Fig. 2:**
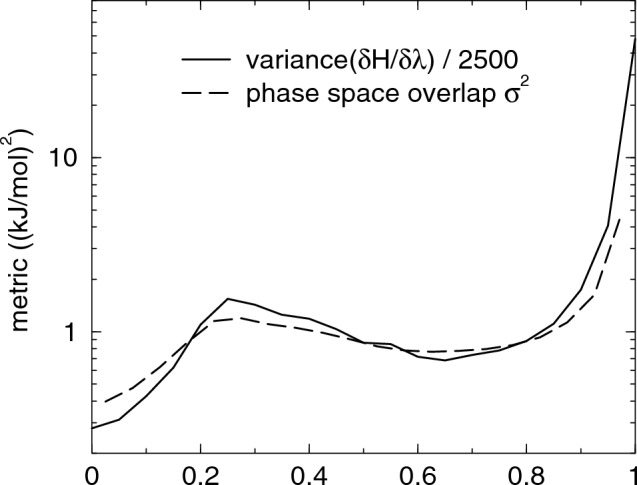
Metrics for the analine dipeptide that do not take into account time correlations: the (scaled) variance of $\partial H/\partial \lambda$ and the phase space overlap $\sigma ^2$ of eq. (11) from [15]

We computed the diffusion metric for AWH and the friction metric for (M)BAR, see Fig. 1. The metric has a local maximum around $\lambda$=0.3, which depends on the value of the $\alpha$ parameter in the soft-core potential, and an absolute maximum at the fully coupled state at $\lambda$=1. The metric is up to an order of magnitude larger for (M)BAR than for AWH. This is expected, as fixing the system along $\lambda$ inhibits relaxation along that dimension, which tends to increase the correlation times. The higher metric with (M)BAR seems to suggest that AWH would be more efficient, but we will see that this is not always the case. To study the effect of time correlations, we compare with two metrics that do not take time correlations into account: the variance of $\partial H/\partial \lambda$, which equals the Fisher-Rao metric, [43] and the phase space overlap $\sigma ^2$ between neighboring $\lambda$-points as defined in eq. (11) from [15]. The results shown in Fig. 2 demonstrate that, for this case, this gives a much flatter metric with only the dip at $\lambda =0$ and the peak at $\lambda =1$ standing out.

To compare the accuracy of the methods, we computed the standard deviation of the computed free energy from a total simulation time of both 4 and 8 ns. Here, and in the two following cases, deviations are computed from an average free energy value obtained from several hundreds of simulations. For AWH we used a flat target distribution, a static optimized target distribution obtained from the metric computed in the flat simulations and a dynamically optimized target distribution. The static optimized target distribution used the same target distribution throughout the whole simulation, but required that the target distribution was obtained beforehand, e.g., from a shorter simulation. In the dynamically optimized case the target distribution was continuously automatically updated to $\pi _\lambda = \sqrt{g(\lambda )}/\mathcal {L}$, when updating the AWH bias, after having left the AWH initial stage. For (M)BAR we ignored the first 100 ps at each $\lambda$-value for equilibration. Using shorter equilibration resulted in systematic errors. The results are reported in Table 1. The efficiency measure $\mathbb {V}$ (eq. (9)) does not vary significantly between simulations; after 8 ns the standard deviation is 10%. The results for AWH largely follow the predictions of $\mathbb {V}$ given by the metric in Eq. (9). There is a clear improvement with both static and dynamic optimization. With (M)BAR the metric is higher, as expected, but in most cases the errors are actually lower than with AWH. Also with (M)BAR the improvement of the optimization is close to the prediction for the 8 ns results. The (M)BAR results for 4 ns show more variation and much larger uncertainties. This is because half the simulation time is spent in equilibration and the sampling time of 190 ps is likely too short for the long correlation times close to $\lambda$=1. We also looked at BAR results with simulation times proportional to the standard deviation of $\partial H/\partial \lambda$. This increases the error slightly compared to the non-optimized case, which demonstrates that it is essential to take time correlations into account.

**Table 1:** Result for the hydration free energy of the alanine dipeptide. Listed are the metric integral $\mathbb {V}$ (eq (9)), the estimate of the improvement factor IF=$\mathbb {V}_\textrm{opt}/\mathbb {V}_\textrm{unif}$ and the root mean square error (RMSE) and IF at 4 and 8 ns. The Fisher-Rao (F.R.) metric optimized results were generated using the variance of $\partial H/\partial \lambda$ as the metric. All AWH values were averaged over 80 simulations, all BAR values over 36 repeats. The RMSE is with respect to the average free energy difference over all simulations for all methods combined. The uncertainties are based on bootstrapping and a confidence interval of one sigma

Method	Num.	Optim.	$\mathbb {V}$	IF est.	4 ns		8 ns	
					RMSE	IF	RMSE	IF
	Points		((kJ/mol)$^2$ ns)		(kJ/mol)		(kJ/mol)	
AWH	21	None	2.0	0.76	1.17 ± 0.09		0.86 ± 0.06	
AWH	21	Diffusion	1.5	0.73	1.02 ± 0.07	0.76	0.76 ± 0.05	0.80
AWH	21	Diff. dyn.			1.05 ± 0.09	0.79	0.71 ± 0.06	0.64
BAR	21	None	5.3	0.68	1.52 ± 0.30		0.70 ± 0.09	
BAR	21	Friction	3.6	0.68	1.01 ± 0.24	0.44	0.61 ± 0.07	0.76
BAR	21	F.R.			1.59 ± 0.25	1.09	0.71 ± 0.10	1.03
MBAR	21	None	5.3	0.68	1.48 ± 0.28		0.69 ± 0.09	
MBAR	21	Friction	3.6	0.68	1.38 ± 0.19	0.87	0.66 ± 0.10	0.91

### Hydration Free Energy of Water

With hydration free energy of water we refer to the free energy of inserting one water molecule in a bulk water solution. The hydration free energy of TIP3P [45] water was calculated by alchemically (de)coupling the interactions of one water molecule to the water molecules surrounding it. The system was included to represent a simple test case, but still requiring sampling of Lennard–Jones as well as Coulomb interactions.

**Fig. 3:**
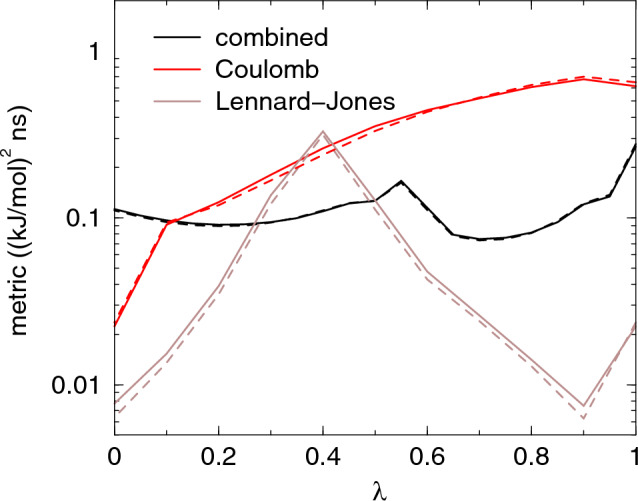
The diffusion metric for the hydration of TIP3P. The solid and dashed lines are the unoptimized and optimized metric, respectively. Note that the metric is plotted on log scale

Two alternative setups of $\lambda$-point distributions were evaluated: modifying Lennard–Jones and Coulomb interactions simultaneously as well as perturbing the interactions sequentially by turning on Coulomb interactions after the Lennard–Jones interactions were fully turned on. In the first case, soft-core potentials were applied for both Lennard–Jones and Coulomb interactions, whereas in the second case they were only used for Lennard-Jones. In both cases there were a total of 21 equidistant $\lambda$ points, i.e., 11 $\lambda$ points each (with one shared) for Lennard–Jones and Coulomb when decoupling them sequentially. The resulting metric is shown for all cases in Fig. 3. The variation of the metric is rather small for the simultaneous coupling and becomes larger when separating Lennard-Jones and Coulomb and coupling them in sequence.

Table 2 shows that the simulations with sequential sampling of Lennard–Jones and Coulomb did not gain anything from optimizing the target distribution. The simulations with simultaneous sampling of the two interaction types had a slight increase of performance (lower RMSE) when optimizing the target distribution. The target value, $-$25.256±0.004 kJ/mol (the standard error of the mean as uncertainty), for the RMSE calculations was based on the mean from 40 sets of simulations with 8 AWH walkers contributing to the same AWH bias, running for 120 ns. The estimated improvement factor, based on the AWH diffusion metric, suggested that there would be a higher gain when decoupling the interactions sequentially. When optimizing the target distribution, the simulations with simultaneous modification of Lennard–Jones and Coulomb interactions were almost as efficient as when modifying the interactions sequentially. However, if there is no specific reason for decoupling the interactions at the same time it is recommended to do it in sequence. With sequential decoupling, $\mathbb {V}$ provides an overestimate of the error, whereas with simultaneous coupling the error is significantly underestimated.

**Table 2:** Results from calculations of hydration free energy of water. The path is either all $\lambda$’s (de)coupled simultaneously (sim.) or Coulomb and Lennard–Jones sequentially (seq.). Listed are the metric integral $\mathbb {V}$ (eq (9)), the estimate of the improvement factor IF=$\mathbb {V}_\textrm{opt}/\mathbb {V}_\textrm{unif}$ and the root mean square error (RMSE) and IF at 1 and 2 ns, from 200 runs, each with 8 communicating AWH walkers. The target distribution optimization was performed during the simulations, after leaving the initial stage. The RMSE was calculated relative to the combined results from 40 sets of simulations each with eight communicating AWH walkers, running for 120 ns. The uncertainties are based on bootstrapping using 5000 samples and a confidence interval of one sigma

Path	Num.	Optim.	$\mathbb {V}$	IF est.	8 $\times$ 1 ns		8 $\times$ 2 ns	
					RMSE	IF	RMSE	IF
	Points		((kJ/mol)$^2$ ns)		(kJ/mol)		(kJ/mol)	
Sim.	21	None	0.11	0.98	0.38 ± 0.02		0.26 ± 0.01	
Sim.	21	Diff. dyn.	0.10	0.97	0.33 ± 0.02	0.76	0.22 ± 0.01	0.73
Seq.	21	None	0.87	0.72	0.24 ± 0.01		0.18 ± 0.01	
Seq.	21	Diff. dyn.	0.60	0.71	0.26 ± 0.01	1.09	0.18 ± 0.01	1.02

### Ligand Mutation

Mutation studies are important in pharmaceutical applications. The most common case is the computation of the difference in binding free energy of two similar drug molecules. Two alchemical free energy calculations are required for this, one in solvent and in the bound state. The challenges for sampling these two processes are similar, so we focus on the solvent case. A mutation study is more complex than the solvation calculation presented before, as it involves making atoms appear and others disappear at the same time. As we will see, this has effects on the diffusion metric.

**Fig. 4:**
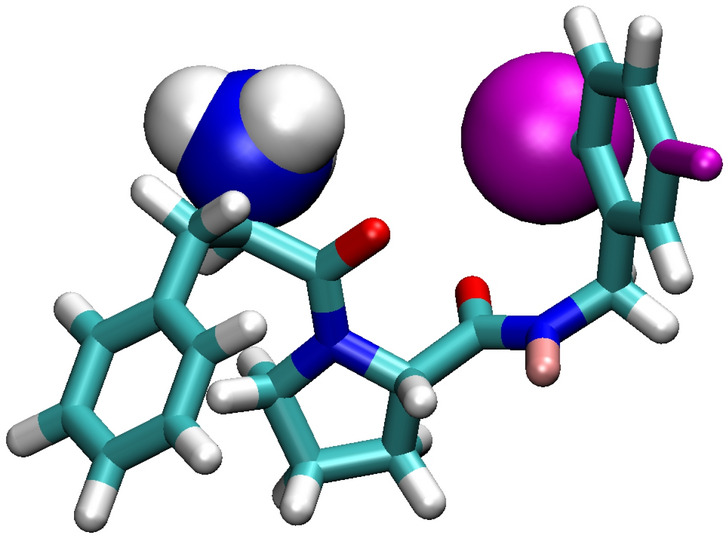
The ligand at $\lambda =0$, corresponding to ligand "6a". Chloride is shown in magenta. At $\lambda =1$, corresponding to ligand "1b", the chloride shown as a sphere is replaced by a hydrogen and the $\hbox {NH}_3$ group uses soft-core interactions at intermediate $\lambda$. Furthermore, some partial charges of nearby atoms change slightly

**Fig. 5:**
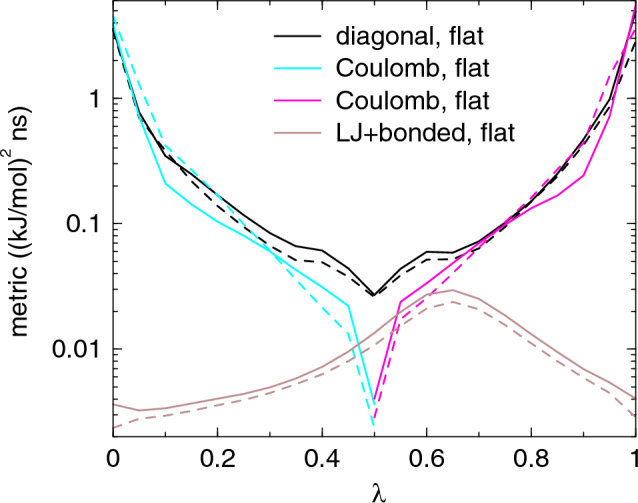
The Diffusion metric for the ligand mutation. The "diagonal" curves are for the path with identical $\lambda$ parameters, the other curves show the three different parts of the improved path with separated legs for Coulomb and other $\lambda$’s. The solid and dashed lines are for flat and optimized sampling, respectively. Note that the metric is plotted on log scale

As a test case we chose "edge 6a - 1b" for the ligand for thrombin [46] for the PMX [47] test set using the GAFF [48] version 2 force field. The molecules consist of 55 and 53 atoms respectively. The main changes are that a chloride atom is replaced by a hydrogen and an $\hbox {NH}_3$ group gets soft-core interactions at intermediate $\lambda$-values (due to the way the mapping tool mapped the two molecules onto a template) when going to 1b (see Fig. 4). The molecules have a net charge of +1, so the system was solvated in box with 993 SPC/E water molecules and 3 $\hbox {Na}^+$ and 4 $\hbox {Cl}^-$ ions. The ligand setup requires constraints on all bonds. After equilibration, production runs of 8 ns were performed. We first look at the metric, see Fig. 5. When modifying all $\lambda$ components simultaneously using 21 $\lambda$-points, we observe very high peaks at $\lambda$=0 and $\lambda$=1, where the metric is a factor 40 higher than in the middle half of the interval. The estimate of improvement optimization would give is rather moderate with a factor 0.64.

**Fig. 6:**
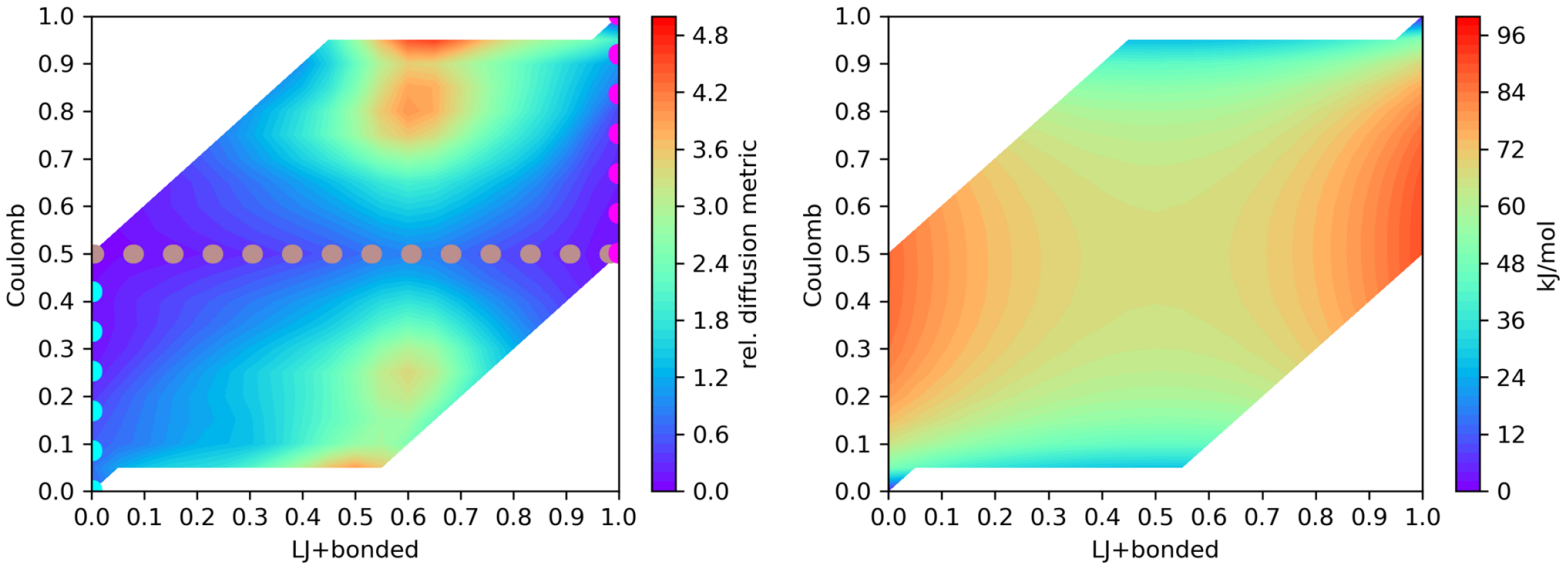
The left panel shows the square root of the determinant of the diffusion metric for the ligand mutation. The scale is relative to the average over the sampled area. The drawn path is the suggested close to optimal path; the colors match those in Fig. 5. To the right the free energy landscape for the ligand mutation is shown

Because of the complexity of this case, it is likely that there could be better paths between the states than the standard approach of coupling all $\lambda$’s simultaneously. The electrostatic interactions are by far the strongest. Thus the Coulomb $\lambda$-component is a good candidate to modify independently. To investigate this, we modified GROMACS [49] to allow separate control and output from individual $\lambda$-components and extended the AWH implementation to act on two alchemical $\lambda$-dimensions. We choose a Coulomb $\lambda$-dimension, whereas we combined all other $\lambda$-dimensions as before. We restricted the sampling to avoid areas where the two $\lambda$-values are very different, as these complicate the sampling and are likely not of interest. In Figure 6 we show the square root of the determinant of the metric tensor. One can see that the landscape is non-trivial. There is a line of low metric precisely at $\lambda _\textrm{Coulomb}$=0.5. This suggests that a better path than the diagonal, which is the standard path, could be to move (nearly) only along $\lambda _\textrm{Coulomb}$ to $\lambda _\textrm{Coulomb}$=0.5 from both endpoints and move along the line in the middle. Note that the actual optimal path would be less angular, but we used completely straight legs to simplify the calculations. Also note that the free energy landscape itself it not at all correlated with the metric. The free energy difference between the end states is only 3.3 kJ/mol, whereas most of the sampled area is between 70 and 100 kJ/mol higher.

To compare the different path and optimizations, we ran AWH simulations for the simultaneous coupling path with 21 points and the separated, sequential, paths with both 21 and 41 points, all with a flat and optimized target distribution. The results are reported in Table (3). For the simultaneous path one can see that the optimization significantly improves the accuracy. The actual improvement is more than three times as large as the estimated improvement factor. When we separate the Coulomb coupling, the metric along the path, shown in Fig. 5 is lower than for the simultaneous path everywhere except for at the end points. But one has to realize that the path is twice as long and therefore the integral of the metric, reported in Table (3) is actually larger. Indeed the error with a flat target distribution is higher than for the simultaneous coupling path. The larger differences in the metric lead to a lower estimate of the improvement factor of 0.35. But the actual improvement is extremely large, with a factor 0.06 for most cases. This shows that very large improvements in efficiency can be achieved, but that this might require a combination of improvements of both the path and the sampling weights. Because of the extreme peaks in the metric present at the end points, we thought that the number of points along the path might have a significant effect on the results. With 21 points the difference in metric between the end points and the direct neighbor is a factor 4 to 5. For the flat target distribution this means that using fewer points might give better results as the weight of the end points is higher. With the optimization more points might be better as one might put too much weight on the end points with the optimized discrete distribution. To check these effects we ran with separate Coulomb path with both 21 and 41 $\lambda$-points. The results do not show significant differences within the statistical error, apart from the optimized case at 8 ns. But when going from 4 to 8 ns, the 21 points results shows much larger improvement than $\sqrt{2}$ while the 41 points case show much smaller improvement. More statistics are needed to draw conclusions here.

**Table 3:** Result for the ligand mutation. The path is either all $\lambda$’s coupled simultaneously (sim.) or sequentially (seq.) with the Coulomb leg separated. Listed are the metric integral $\mathbb {V}$ (eq (9)), the estimate of the improvement factor IF=$\mathbb {V}_\textrm{opt}/\mathbb {V}_\textrm{unif}$ and the root mean square error (RMSE) and IF at 4 and 8 ns. All AWH values were averaged over 80 simulations, all (M)BAR values over 20 repeats. The RMSE is with respect to the average free energy difference over all simulations for all methods combined. The uncertainties are based on bootstrapping and a confidence interval of one sigma

Method	Path	Num.	Optim.	$\mathbb {V}$	IF est.	4 ns		8 ns	
						RMSE	IF	RMSE	IF
		Points		((kJ/mol)$^2$ ns)		(kJ/mol)		(kJ/mol)	
AWH	Sim.	21	None	3.6	0.64	3.4 ± 0.5		3.2 ± 0.5	
AWH	Sim.	21	Diffusion	2.1	0.60	1.7 ± 0.2	0.21	1.6 ± 0.3	0.23
AWH	Seq.	21	None	20.6	0.27	4.9 ± 0.5		4.1 ± 0.4	
AWH	Seq.	21	Diffusion	4.9	0.27	1.2 ± 0.1	0.06	0.7 ± 0.1	0.03
AWH	Seq.	41	None	8.4	0.41	4.7 ± 0.4		4.1 ± 0.5	
AWH	Seq.	41	Diffusion	3.2	0.35	1.1 ± 0.1	0.06	1.0 ± 0.1	0.06
BAR	Sim.	21	None	3.9	0.39	5.6 ± 0.9		4.8 ± 0.9	
BAR	Sim.	21	Friction	1.5	0.39	5.3 ± 1.0	0.90	3.7 ± 0.7	0.57
MBAR	Sim.	21	None	3.9	0.39	1.5 ± 0.2		1.3 ± 0.2	
MBAR	Sim.	21	Friction	1.5	0.39	1.0 ± 0.2	0.45	0.7 ± 0.1	0.29

For comparison, we also performed a small number of (M)BAR calculations for the simultaneous coupling path with 21 points. Unlike the alanine dipeptide case, BAR shows larger errors than AWH. MBAR, on the other hand, performs significantly better than AWH when simultaneously coupling the Coulomb interactions. These MBAR results reach similar accuracy as AWH with separated Coulomb coupling. This indicates that there is significant ensemble overlap between some non-neighboring $\lambda$-values. For the ligand mutation calculations, $\mathbb {V}$ often provides a good estimate of the error, only the sequential coupling with optimization shows lowers errors than expected.

## Conclusions

The $\lambda$-coupling approach is a powerful tool for computing free energy differences using molecular simulations. By design, this approach requires choosing a path between the end states, as well as a parametrization and sampling distribution. Although the community has realized that the choice of path can have a strong effect on the efficiency of free energy calculations, efforts have mainly been focused on optimizing the distribution of $\lambda$-points. The parametrization, or conversely, distribution of $\lambda$-points has become a standard consideration. But optimizing the distribution of $\lambda$-points is most often done empirically, usually by adding a few more points close to the fully coupled state(s).

We have demonstrated here that with a suitable metric it is possible to systematically optimize both the path and target distribution in alchemical free energy calculations. For expanded ensemble simulations, in our case, AWH, which carry out a diffusive random walk along the $\lambda$-coordinate, such a metric is the diffusion metric defined in Eq. (7), while for methods based on independent simulations carried out for a range of fixed $\lambda$-values, the friction metric in Eq. (6) is appropriate. Both metrics are defined by integrated time correlation functions, and are thus able to identify and compensate for the variations in the correlation time along the path. The relevance of these metrics follows from a direct, although approximate, relation to the variance of the estimate $\overline{\Delta G}$. The variance asymptotically decays as $1/\tau$ with simulation time $\tau$, and a useful measure for the efficiency is provided by $\mathbb {V} \approx \tau \textrm{Var}\ \overline{\Delta G} / 2$. The quantity $\mathbb {V}$ defined in Eq. (9) often gives a rough estimate of the accuracy of the computed free energy, although in some cases the estimate differs significantly from the empirical one.

We find that distributing the sampling according to the square root of the metric almost always improves the accuracy of the results. Only for the case of water with sequential Lennard–Jones/Coulomb coupling did we not observe a significant difference. One can compute a theoretical estimate of improvement the optimization will provide, but in several cases the actual improvement differs quite significantly from this estimate. In the worst cases we observed no improvement, or only an improvement of a few percent. But in some cases we saw an improvement in the efficiency of more than an order of magnitude. An essential aspect of the diffusion metric is that it takes time correlations into account. For the solvation of the alanine dipeptide we have shown that sampling according to the Fisher-Rao metric, that disregards time correlations, does not improve the accuracy. In AWH calculations, the diffusion metric can be computed on the fly and the target distribution can be optimized after the leaving the initial phase. We have demonstrated that this provides automated tuning and improvement of the efficiency which is at least as good as static optimization. This means that the optimization comes "for free", in the sense that the user only has to toggle optimization on. One could even consider turning on the optimization by default when using AWH for alchemical calculations, but the set of three test cases presented here is too small to make that decision.

And finally, the metric enables optimization of the path itself. As the metric is defined for any number of dimensions, one can, in theory, sample a multidimensional space and choose the optimal path, i.e. the path with the lowest metric integral. We have shown an example for the case of a ligand mutation. Here the 2D metric landscape shows a straightforward improvement of the path by first moving to the half-way point along the Coulomb $\lambda$ component. This improves the efficiency by a factor of 2 to 4 compared with the standard path of using a single $\lambda$-component. Combined with the optimization of the target distribution, the total efficiency improvement is a factor 16. What would be more powerful and convenient is a partially automated improvement of the path. This requires a path search in the metric space. For low dimensional spaces this is tractable. It is important to realize that the $\lambda$-coupling approach allows for infinitely many ways to vary $\lambda$, so it is questionable if it is even theoretically possible to find the optimal path. But in practice the Lennard–Jones and Coulomb interactions often dictate what states the system will sample. Thus we expect that optimizing the path in this 2D space can often bring significant efficiency improvements.

## Methods

The molecular dynamics simulations were performed using GROMACS [49]. Except where noted otherwise, all simulations used Beutler [20] soft-core interactions for both Lennard–Jones and Coulomb interactions using the Lennard–Jones soft-core distance also for Coulomb, [21] with a power of 1 and $\alpha$ = 0.5. All simulations used smooth particle-mesh Ewald [50] for electrostatics with a relative tolerance at the cut-off of 10$^{-5}$. The BAR analyses were performed with the GROMACS BAR tool. The MBAR analyses were performed with the ${\texttt {pymbar}}$ and ${\texttt {alchemlyb}}$ packages. No resampling for statistical inefficiency were performed. The RMSEs were calculated by bootstrapping the results from the repeated simulations.

### Hydration Free Energy of the Alanine Dipeptide

The AMBER99 [51] force field was used in combination with the SPC/E water model. [52] The non-bonded interactions were cut off at 1 nm. Temperature was set to 298 K by using a stochastic dynamics integrator [53] (also referred to as a velocity Langevin dynamics integrator) with a time step of 2 fs and with a time constant $\tau$ of 1 ps (corresponding to a friction constant of 1 $\hbox {ps}^{-1}$). The pressure was set to 1 atm and controlled using an isotropic stochastic cell rescaling barostat [54] with a time constant of 2 ps and a compressibility of 4.5e$-$5 $\hbox {bar}^{-1}$. For AWH we used a diffusion of 0.01 $\hbox {ps}^{-1}$ and an initial error of 100 kJ/mol to set the initial update size. Between 80 and 100 simulations were run for each setup. For comparison, we also ran BAR with 36 repeats, where we disregarded the first 100 ps at each $\lambda$-point for equilibration.

### Hydration Free Energy of Water

In these simulations non-bonded interactions had a cutoff of 1.2 nm, switching Lennard–Jones interactions to 0 between 1.0 and 1.2 nm. Long-range electrostatic interactions were treated with PME. Temperature was set to 298.15 K by using a stochastic dynamics integrator [53] with a time step of 2 fs and with a time constant $\tau$ of 2 ps (corresponding to a friction constant of 0.5 $\hbox {ps}^{-1}$). The pressure was set to 1 atm and controlled using an isotropic stochastic cell rescaling barostat [54] with a time constant of 10 ps and a compressibility of 4.5e$-$5 $\hbox {bar}^{-1}$. The simulations using a sequential (de)coupling schedule used a soft-core potential for the Lennard-Jones interactions, but not for Coulomb. In the simulations that (de)coupled the interactions simultaneously the Coulomb interactions were also treated using soft-core potentials, with the same settings for both interaction types. The simulations were performed with and without automatic optimization of the target distribution, after leaving the AWH initial stage. 200 simulations each with 8 communicating walkers were run for 2 ns. The input AWH diffusion coefficient was set to 0.001 $\hbox {ps}^{-1}$ and the initial error to 10 kJ/mol.

### Ligand Mutation

The general AMBER force field [48] version 2 was used in combination with the SPC/E water model. The non-bonded interactions were cut off at 1 nm. A leap-frog integrator was used with a time step of 2 fs. Temperature was set to 298 K by using a velocity rescale thermostat [55] with a time constant $\tau$ of 1 ps. The pressure was set to 1 atm and controlled using an isotropic stochastic cell rescaling barostat [54] with a time constant of 2 ps and a compressibility of 4.5e$-$5 $\hbox {bar}^{-1}$. For AWH we used a diffusion of 0.005 $\hbox {ps}^{-1}$ and an initial error of 50 kJ/mol to set the initial update size. We ran between 80 and 100 simulations for each setup. For comparison, we also ran BAR with 20 repeats, where we disregarded the first 100 ps at each $\lambda$-point for equilibration. To enable AWH sampling along two $\lambda$-components, we modified GROMACS [49] to allow control and feedback of individual $\lambda$-components. This code is experimential and is available from the GROMACS gitlab repository [56].

### Automatic $\lambda$ Point Distribution Optimization During AWH Simulations

In GROMACS the option to automatically update the target distribution, based on the AWH diffusion metric, was added. After the leaving the initial stage of AWH the target sampling of each point is set to the square root of the determinant of the diffusion metric, whenever the AWH bias is updated, but no more frequently than every 100 MD simulation steps. This feature will be added in GROMACS version 2024, and is currently available for testing from the GROMACS gitlab repository [57].
